# Rare complication – skin atrophy – after systemic conservative therapy of infantile hemangioma

**DOI:** 10.1186/s12887-024-04638-w

**Published:** 2024-02-23

**Authors:** Konstantine Chakhunashvili, Eka Kvirkvelia, Natia Todua, Davit G. Chakhunashvili

**Affiliations:** 1 The University of Georgia, Tbilisi, Georgia; 2 Children’s Clinic after I. Tsitsishvili, Tbilisi, Georgia; 3 Department of Gynecology, Caucasus University, Tbilisi, Georgia; 4 Todua Clinic, Head of The Dermatology Department, Tbilisi, Georgia; 5 Abuladze Georgian-Italian Clinic, Tbilisi, Georgia; 6 Department of Pediatrics, Alte University, Tbilisi, Georgia

**Keywords:** Infantile hemangioma, Propranolol, Skin atrophy

## Abstract

**Background:**

Hemangiomas, also called infantile hemangiomas (IH) or hemangiomas of infancy are the most frequently seen benign vascular tumors of infancy. Different types of hemangiomas are described in the literature. The current approach is to assess the risk and, if needed, first line treatment is to initiate systemic propranolol.

**Case presentation:**

A 3-month-old Caucasian female patient was brought as an outpatient. The main complaint was an infantile hemangioma in the facial area, which as per the parents’ story appeared within a week of birth like a small reddish line and it rapidly grew. Systemic propranolol was proposed as a first-line treatment and the adverse effects were explained. The parents, afraid of the side effects, wanted to explore other possibilities such as topical timolol, however, since it had no effect, propranolol was initiated in the end. Hemangioma was completely reduced in size; however, a skin defect was detected. As per the dermatologist’s counsel, topical cream was initiated. The skin defect was reduced but not fully healed. The child is still being monitored periodically.

**Conclusion:**

After successful treatment of hemangioma, we identified a skin defect, which was very similar to steroid-induced skin atrophy. However, we cannot attribute this to a single factor. The only thing that can be concluded is that the subject needs a thorough studying, since rate of infantile hemangioma is high, and pediatricians need a clear management strategy of how to approach skin atrophy after successfully treating the hemangioma itself.

## Introduction

Hemangiomas, also called infantile hemangiomas (IH) or hemangiomas of infancy are the most frequently seen benign vascular tumors of infancy [1]. Different types of hemangiomas are described in the literature; While infantile hemangiomas evolve later in infancy, congenital hemangiomas are already present at birth [1]. Around 80% of Infantile hemangiomas effect face and neck region [2]. Cycle of IHs consists of rapid proliferation phase, plateau phase and slow involution phase. At around 9 months maximum size is already achieved and by the age of 4 years regression process is completed in 90% of children [1]. Family history of IH, multiple gestation, preterm birth, female gender, low birth weight and progesterone therapy are the risk factors of IHs [2]. Infantile hemangiomas, based on their distribution can be classified into different types: segmental, focal, multifocal and indeterminate [2]; Segmental IHs are more prone to complications, thus require therapy in majority of cases. According to morphology, IHs are classified into deep, superficial and mixed types [3]. Pathogenesis of IHs is not well-studied, but is thought to develop after endothelial cell proliferation and cellular hyperplasia [4].

## Case presentation

### Patient information

**Fig. 1:**
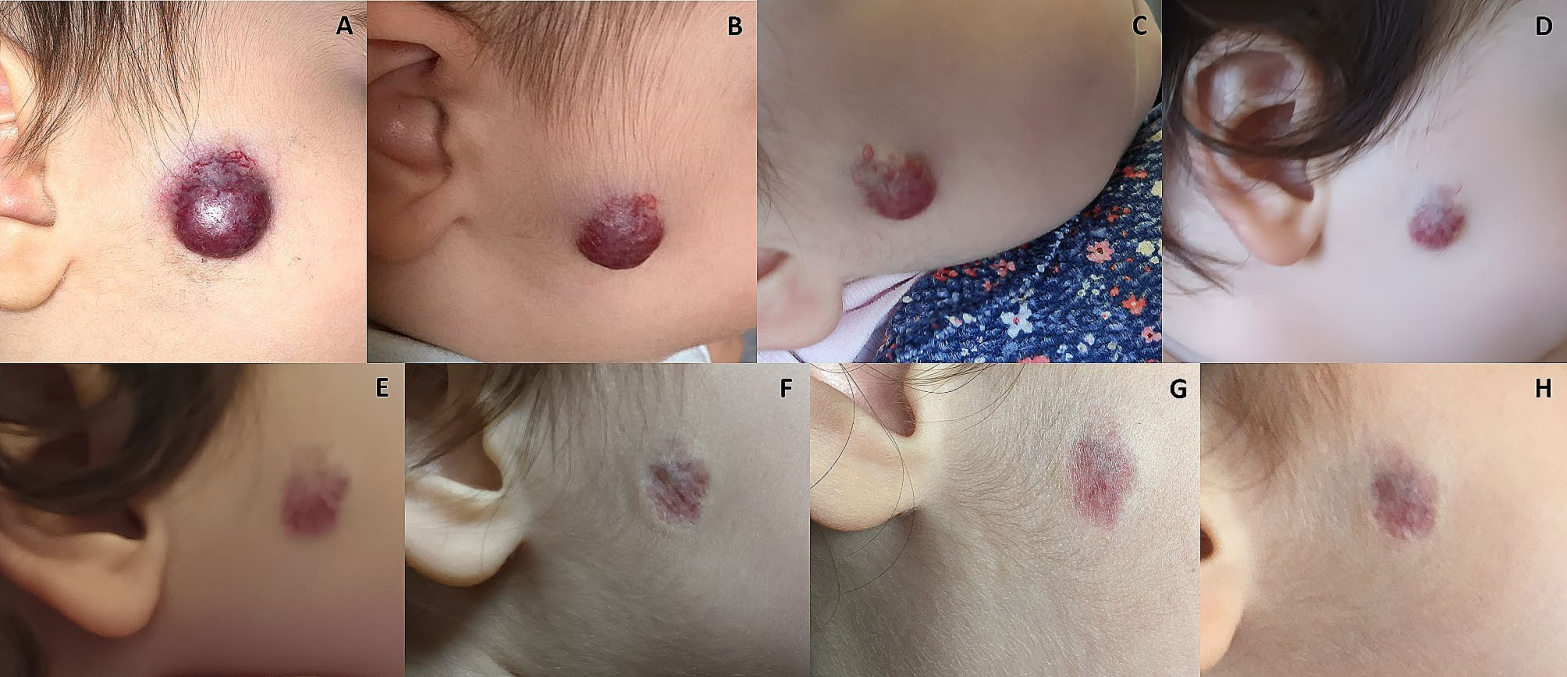
(**A**) before initiation of propranolol treatment. (**B**) 16 days after initiation of the treatment. (**C**) 2 months after initiation. (**D**) 6 months after initiation. (**E**) Right before the dose taper was initiated (11 months after initiation of propranolol). (**F**) Propranolol treatment finalized (13 months after initial dose) and topical cream initiated. (**G**) 5 months after topical cream started and propranolol treatment had been finalized. (**H**) 10 months after topical cream started and propranolol treatment had been finalized

A 3-month-old Caucasian female patient was brought as an outpatient. The main complaint was an infantile hemangioma in facial area (Fig. 1), which as per parents’ story appeared within a week of birth like a small reddish line and it rapidly grew.

### Timeline and diagnostic assessment

On the day of the visit in December 2021, the patient had been thoroughly examined not to miss any mass lesions to other organs and organ systems. Physical examination yielded that the child’s growth and development were normal, and no signs of other organ involvement were detected. Oral propranolol had been offered as a first line of treatment, however, as the adverse events were explained, they resorted to topical treatment with timolol for a month to check the effect. As it had little to no effect after 1 month follow-up visit, in early January 2022, oral propranolol had been initiated and after a month the lesion started to shrink. By November of 2022 the hemangioma was completely gone, and we started to taper the dosage, treatment was stopped in January 2023.

**Fig. 2:**
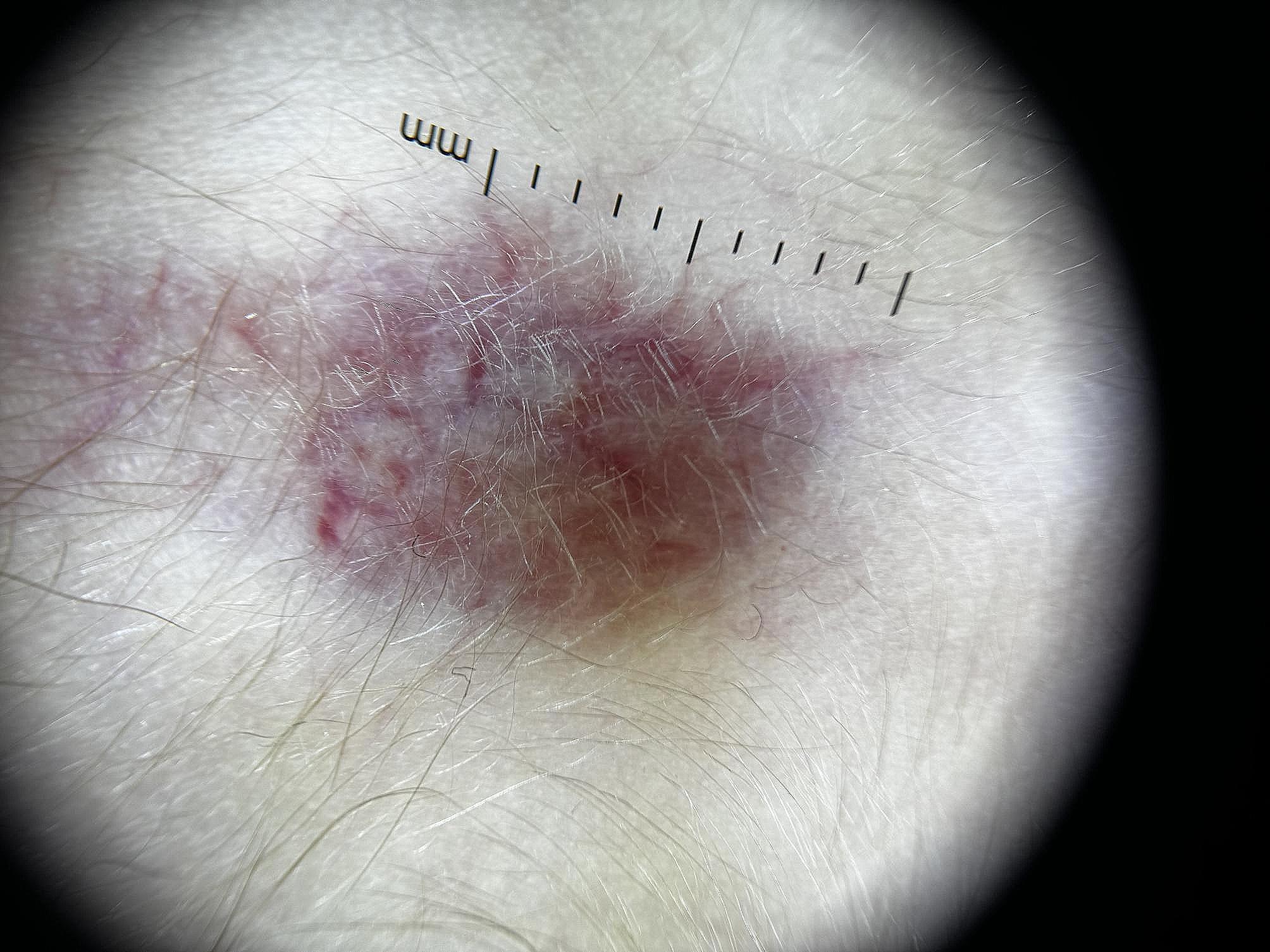
A dermoscopy image (magnification = X10) of the lesion shows asymmetry, irregular borders, homogeneous surface with pinkish-skin-colored areas, irregular vascular pattern (examination was performed 11 months after propranolol treatment had been finalized)

By the end of the treatment, we could not detect hemangioma, but there was a visible skin defect, like steroid-induced skin atrophy (Fig. 1). To address this complication, we searched for counsel from a dermatologist, who suggested a topical treatment. The treatment was initiated in January 2023, and it yielded a positive effect – the defect has been reduced and the area of the skin atrophy is slowly starting to diminish (Figs. 1 and 2).

### Therapeutic intervention

Initial topical therapy chosen by parents was timolol, which was applied to the lesion 3 times a day.

Oral propranolol was started after timolol was deemed ineffective; The dosage was slowly increased. 0.5 mg/kg/day was given for a week, on the 8th day it was increased to 1 mg/kg/day, on the 12th day – 1.5 mg/kg/day and on the 16th day final target dosage of 2 mg/kg/day was achieved. Daily dosage was split 3 times and medication was administered at 8:00, 16:00, and 24:00, 15 min after feeding. Up to 6 months dosage was corrected every month and every 2 months after 6 months of age.

For atrophied skin, the dermatologist suggested treatment with Scarvis, which contains glycerol and dimethicone, 3 times a day.

### Follow-up

Currently follow-up is done every 6 months via outpatient visit and online every month.

## Discussion

Infantile Hemangiomas still remain to be a challenge for the medical field, constantly raising questions about pathogenesis, risk factors, prognosis, evolution and management. Even though IHs are mostly harmless and „wait-and-see“ approach is reasonable (due to self-involution growth pattern), there are circumstances when intervention is crucial and compulsory [4]. The treatment of IH depends on different factors, like localization, stage and distribution type of the lesions, hemangioma type, presence of ulceration, systemic involvement, level of psychosocial condition of the child or parent [3]. Location of hemangiomas plays an important role in the selection of treatment approach. Any life threatening (airway) or function threatening (eyelid, periocular, lip, ear, nasal or ano-genital region) lesions are at increased risk of complications and early specific therapeutic intervention is needed [3, 5]. Active non-intervention tactic can be applied in majority of the remaining cases (especially when superficial, small, are covered with hair or cloth, small) which includes active observation of the infant during first few months and the education of the parent about course of the disease, intervention methods, complications and anticipatory guidance [3, 6]. When decision about the therapeutic intervention of IH is made, pre-treatment work-up should be executed. Before starting therapy via propranolol, children should be assessed for certain contraindications (propranolol allergy, heart failure, sinus bradycardia, bronchial asthma, hypotension, hypoglycemia, heart block, hypotension) [3]; Blood sugar levels, blood pressure and heart rate should be monitored and electrocardiogram should be performed as well. Pre-treatment investigations before initiation of steroid therapy comprises of monitoring anthropometric parameters and blood pressure, ruling out of primary immunodeficiency and active infection, performing of CBC, serum biochemistry tests, stool microscopy and chest x-ray [3].

Since 2008, the approach by Laute-Labreze, which includes the use of Propranolol as a treatment option of infantile hemangiomas, became first-option therapy in most of the cases because of its few adverse effects and favorable outcomes [6].

The serious side-effects of propranolol treatment such as dyspnea, hypoglycemia and bradycardia are well known [7–10]. There also has been an association with certain medical skin conditions like psoriasis and beta-blockers [11]. It is also known that treatment with local injections can end in soft tissue atrophy [12]. We could not identify literature that implicated systemic beta-blockers for skin atrophy lesions. The skin lesions, such as skin atrophy, ulceration and hyperpigmentation have been reported after pulsed dye laser treatment as well [13].

Nowadays, skin atrophy is a huge challenge in dermatology. For example, there are many procedures that can be used in the management of atrophic acne scars: Resurfacing procedures (Chemical peeling, dermabrasion), lifting procedures (subcision, fillers), excisional techniques (punch grafting, elliptical excision) and others (skin needling, combinations techniques etc.) [14]. Skin atrophy is one of the complications of topical corticosteroid use as well [15]; Although it should resolve on its own after cessation of therapy, which can take months [15].

Topical and systemic beta-blockers have been associated with good results in regards to treatment of atrophic scars, keloids etc. [16]. 

One of the interesting components of the case is that we are unsure what was the thing that triggered development of the skin defect: was it systemic administration of propranolol, was it somehow linked to initial topical timolol solution, or was it linked to involution of the lesion, which due to abrupt borders of the IH started to form such a sequela.

There have been multiple reports indicating that involution of IH may lead to various types of lesions, including telangiectasia, fibrofatty tissue, and anetodermic skin. However, the specific factors contributing to this development are still not fully understood [17, 18]. Two studies have focused on the complications observed in a total of 358 patients: 187 patients who went untreated [17] and 171 patients who received treatment (oral propranolol) [18]. Among the untreated patients, an alarming 54.9% developed significant sequelae, while only 26.9% of the treated patients experienced such complications. This difference is considered statistically significant (chi-squared − 30.7, DF − 1, *P* < 0.0001), indicating that there is a lower chance of complications when using oral propranolol as a treatment. It is important to note, however, that based solely on this data, we cannot confirm or rule out oral propranolol as a definite causative agent for the observed outcomes, including our lesion at hand. Further research and investigation are needed to fully understand the relationship between oral propranolol and the development of these complications.

The other thing that makes us curious is the following: did the lesion start to heal itself or did the topical cream make a difference? The depth and size of the defect was clearly reduced (Figs. 1 and 2), however, since this was a single case and we did not compare it to a control, it would not be scientifically correct to estimate one or another. One thing we can say for sure is that further research is warranted, so that clinicians know what the appropriate management strategies for a lesion like this are, since the incidence of hemangioma is known to range from 0.97 to 1.97% [19].

## Conclusion

After successful treatment of hemangioma, we identified a skin defect, which was very similar to steroid-induced skin atrophy. However, we cannot attribute this to a single factor – systemic propranolol, topical timolol, or the size of the lesion itself. The size of the lesion was reduced over time; however, it would be extremely imprudent to link improvement with the topical cream that was used. The only thing that can be concluded is that the subject needs a thorough studying, since rate of infantile hemangioma is high, and pediatricians need a clear management strategy of how to approach skin atrophy after successfully treating the hemangioma itself.

## Data Availability

[REAGENTS/TOOLS/MATERIALS] generated in this case report are available from the corresponding author upon request.
